# The molecular characteristics of gastric cancer patients living in Qinghai-Tibetan Plateau

**DOI:** 10.1186/s12876-022-02324-8

**Published:** 2022-05-14

**Authors:** Ling Yuan, Shilong Chen, Yongcui Wang, Yingcai Ma

**Affiliations:** 1 Medical College, Soochow University, Suzhou, 215123 Jiangsu China; 2 Department of Gastroenterology, Qinghai Provincial People’s Hospital, Xining, 810007 Qinghai China; 3 Key Laboratory of Adaptation and Evolution of Plateau Biota, Northwest Institute of Plateau Biology, Chinese Academy of Sciences, Xining, 810008 Qinghai China; 4 Institute of Sanjiangyuan National Park, Chinese Academy of Sciences, Xining, 810008 China; 5 Qinghai Provincial Key Laboratory of Crop Molecular Breeding, Northwest Institute of Plateau Biology, Chinese Academy of Sciences, Xining, 810008 China

**Keywords:** Gastric cancer, Qinghai-Tibetan plateau, Molecular characteristics, The mechanism of cancer, Cancer targeted therapeutics

## Abstract

Gastric cancer, or stomach cancer, that originates in the inner lining of the stomach, was the fifth most common cancer and the fourth mortality globally, with over one million new cases in 2020 and an estimated 769,000 deaths. The molecular characteristics of gastric cancer has been complicated by histological and intratumor heterogeneity. The incidence of gastric cancer shows wide geographical variation. As the largest and highest region in China, Qinghai-Tibetan Plateau is one of the important global biodiversity hotspots. Here, we collect tumour and paired normal bio-samples from 31 primary gastric cancer patients from Qinghai Provincial People’s Hospital, and discuss the molecular characteristics for gastric cancer patients living in plateau. They have more single nucleotide polymorphisms (SNP) located in chromosome 7 with C → T and G → A as the most common alteration types, barely share the cancer driver genes with western patients, and have no significant differences in various Chinese nation. These characteristics offers a great opportunity to further understanding the divergent mechanism of gastric cancer, increase the efficacy for diagnosis and prognosis, finally lead the optimal targeted therapeutics.

**Supplementary Information:**

The online version contains supplementary material available at 10.1186/s12876-022-02324-8.

## Introduction

Gastric cancer is one of the most common gastrointestinal malignancies in the world. It is a malignant tumour originating from the muscosal epithelical cells of the gastric. Gastric cancer can occur in any part of the stomach, with the lesser curvature of the gastric antrum and the anterior area of the pylorus the most common, followed by the fundus of the stomach from the cardia [1]. Most gastric mucosal lesions are gradually developed into gastric cancer by atrophy, intestinal metaplasia, low-grade intraepithelial neoplasia and high-grade intraepithelial neoplasia. Early gastric cancer has a good prognosis, with a 5-year survival rate of more than 90%, while advanced gastric cancer has a poor prognosis, with 5-year survival rate of less than 30% [2–4]. In recent years, the overall incidence of gastric cancer has decreased, but it is still the fifth most common tumour and the second most lethal cause of cancer in the world. About 950000 new cases of gastric cancer are diagnosed globally every year, and about 700000 patients die from it [5]. In 2020, gastric cancer was responsible for over one million new cases and an estimated 769,000 deaths (equating to one in every 13 deaths globally), ranking fifth for incidence and fourth for mortality globally [6]. Previous studies have shown that the incidence of gastric cancer varies by region [7], with more than 50% of new cases occurring in developing countries.

The incidence of gastric cancer in China ranks third in the world, and the mortality rate among malignant tumors ranks first [7]. The morbidity and mortality of different regions in China also have obvious differences, among which northwest China and northeast China are highly affected. Northwest China, such as Qinghai, Ningxia, Gansu, has high mortality, while Qinghai has the highest mortality [8]. Qinghai province is located in the Qinghai-Tibet Plateau (QTP). QTP has a complex geological history, and it is a common understanding that the central plateau uplifted first and formed the `proto-QTP’ as early as 40 Mya, followed by outward extensions in early Miocene [9–12]. The agricultural and pastoral areas in Qinghai are vast, with difficult natural conditions, poor nutrition, relatively weak sanitary conditions and awareness, and high Helicobacter pylori (HP) infection rate, leading to high incidence of gastric cancer. The diagnosis rate of early gastric cancer in China is only 5%-20% [5]. Qinghai has a low detection rate of early gastric cancer and a high mortality rate due to its special low-oxygen regional environment and dietary habits. Therefore, we analyzed the clinical and molecular characteristics of gastric cancer patients living in Qinghai Province, in order to better understand its pathogenesis, so as to know the individualized treatment.

Here, for better understanding the molecular mechanism of gastric cancer patients living in QTP and suggest the targeted therapeutic strategies specially designed them, we collected tumours and paired normal bio-sample from 31 gastric cancer patients from Qinghai Provincial People’s Hospital, discussed their unique molecular characteristics, and predicted the specific therapeutic drugs based on adapted kernel-based machine learning method.

## Method

### Tumor specimens and their paired normal bio-samples

After receiving informed consent, tumor and paired normal bio-samples were obtained from patients undergoing surgery at Qinghai Provincial People’s Hospital. This work was performed in compliance with all relevant ethical regulations for research using tumor and paired normal specimens. The fresh frozen tissues were delivered to sequencing company, Frasergen (Additional file 1: Table S1), to capture the exonic DNA fragments and perform the whole exome sequencing. All methods were performed in accordance with the relevant guidelines and regulations for research using human specimens.

### The pharmacogenomics data that used for training drug response learning model

The pharmacogenomics used to train the prediction model came from the drug response data on cancer cell lines, which was deposited in GDSC [31]. The mutation profile for genes across cancer cell lines and chemical structures for anti-cancer drugs were used to represent cancer cell lines and drugs, respectively.

### Whole exome sequencing and mutation calling

The Illumina HiSeq 2000 instrument was applied for whole exome sequencing (WES), which generated 2 × 150 base paired-end reads. FASTQ files were aligned to the human genome assembly (hg38) via Burrows–Wheeler Aligner (BWA) [13]. Before further analysis, the initially aligned BAM files were pre-processed that sorted, removed duplicated reads, locally realigned reads around potential small indels, and recalibrated base quality scores via SAMtools [14] and Picard (https://broadinstitute.github.io/picard/). The single nucleotide polymorphisms (SNP) was detected through the Genome Analysis ToolKit (GATK) [15] and annotated via ANNOVAR [16]. The duplicated and low-quality SNPs were removed before annotation.

### Genome coverage, somatic mutation, and gene fusion analysis

The coverage per-base was calculated from genomecov function in bedtools package [17] based on preprocessed BAM files. The coverage at gene level was obtained based on the human genome (hg38) annotation file. The somatic mutation was obtained by using SNPs from paired normal bio-samples as a reference. The gene fusion analysis was performed via FusionMap [18] based on FASTQ files.

### The model for prediction of the effective clinical drugs

We applied our previously prediction model, an adapted kernel-based learning model to predict the effective clinical drugs for cancer patients [19]. Specifically, a bipartite graph framework under the assumption that drugs with similar chemical properties should have similar treatment outcomes, was introduced to represent the relationship between cancer cell and anti-cancer drug [19]. An adapted kernel method was proposed to construct similarity matrix based on different types of features. That is, the cancer genomic data (such as mutation, expression, et al.) and chemical properties were applied to construct kernel-based similarity matrices between cancer cells and anti-cancer drugs. The three classification models, random forest (RF), support vector machine (SVM), and deep learning network (DN) were then applied on these kernel-based similarity matrices, separately, to predict the effective clinical drugs for cancer patients. The RF, SVM and DN model were implemented via `randomForest’ R package with default parameters, LibSVM [20] in ‘e1071’ R package with RBF kernel function, and the `h2o’ R package with default parameters, respectively. The penalty parameter and the RBF kernel parameter were optimized by the grid search approach with fivefold cross-validation. The area under the ROC curve (AUC) [21] was introduced as the evaluation criteria to assess the performance of classification model.

### Identification of cancer driver gene

The MaxMIF [22], which was reported to outperform the existing state-of-the-art methods (including MUFFINN [23], MuttSig2 [24], MutSigCV [25], et al.) on TCGA pan-cancer datasets, was introduced to distinguish the cancer driver genes from the passenger genes. MaxMIF integrated the somatic mutation data and molecular interaction data by a maximal mutational impact function. The protein–protein interaction (PPI) network deposited in HumanNet v2 [26] was introduced to represent the molecular interactions.

## Results

### Clinical characteristics

**Fig. 1:**
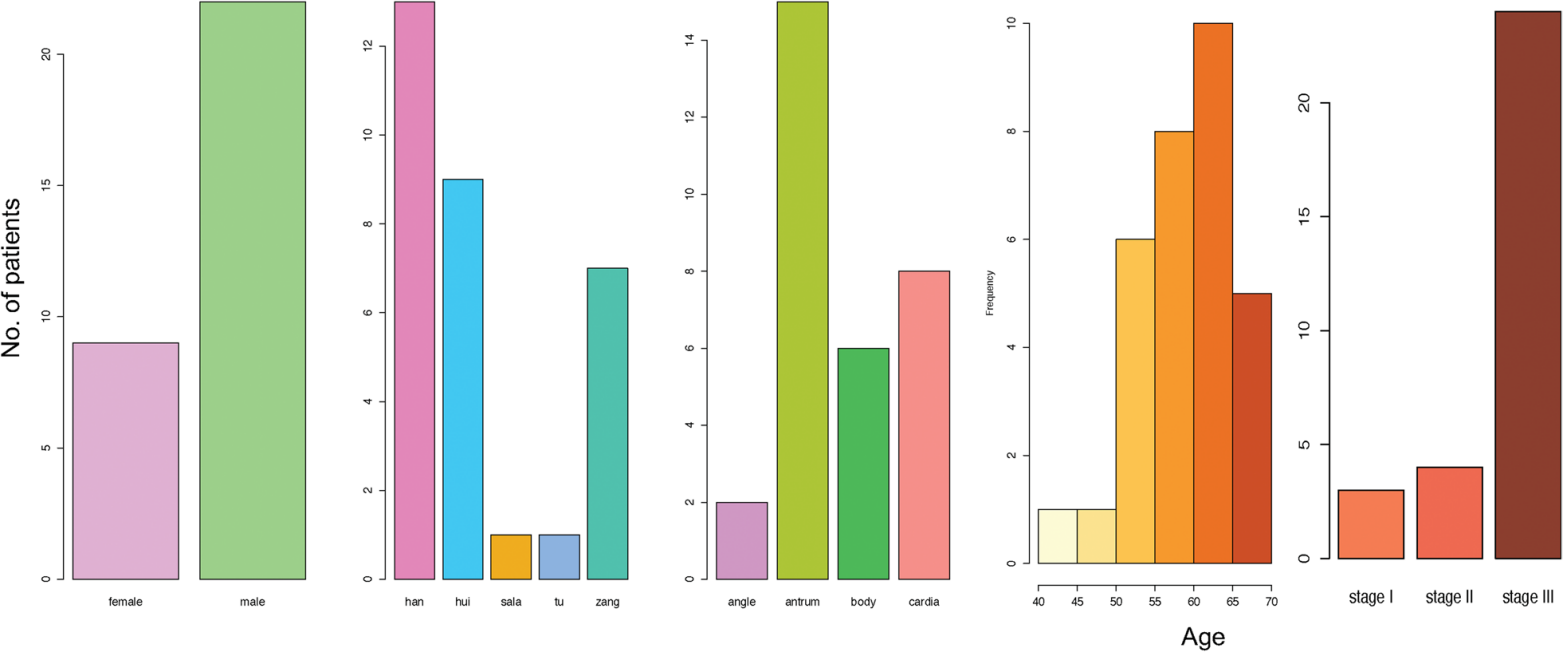
The clinical characteristics for gastric adenocarcinoma patients collected from Qinghai Provincial People’s Hospital. The clinical information includes the gender, ethnic group, tumor location, and initial diagnosis age for gastric adenocarcinoma patients

We obtained gastric primary tumor tissue (fresh frozen) from 31 patients not treated with prior chemotherapy or radiotherapy. All tumor tissues are adenocarcinoma. The clinical information, including the initial diagnosis age, the gender, the location of primary tumor tissue, tumor TNM stage, and the patients’ nation, was shown in Fig. 1. Most of patients were male (22/31), the initial diagnosis age was from 40 to 70 years old, and most of these patients were from 55 to 65 years old. Comparing with the western patients in terms of initial diagnosis age, plateau patients were younger (western patients are around sixty) [27]. Furthermore, most of patients (24/31) got stage III tumors. The differences in clinical properties indicate the unique properties for these patients living in QTP, meaning they may need the specific treatment strategy.

### Molecular characteristics

**Fig. 2:**
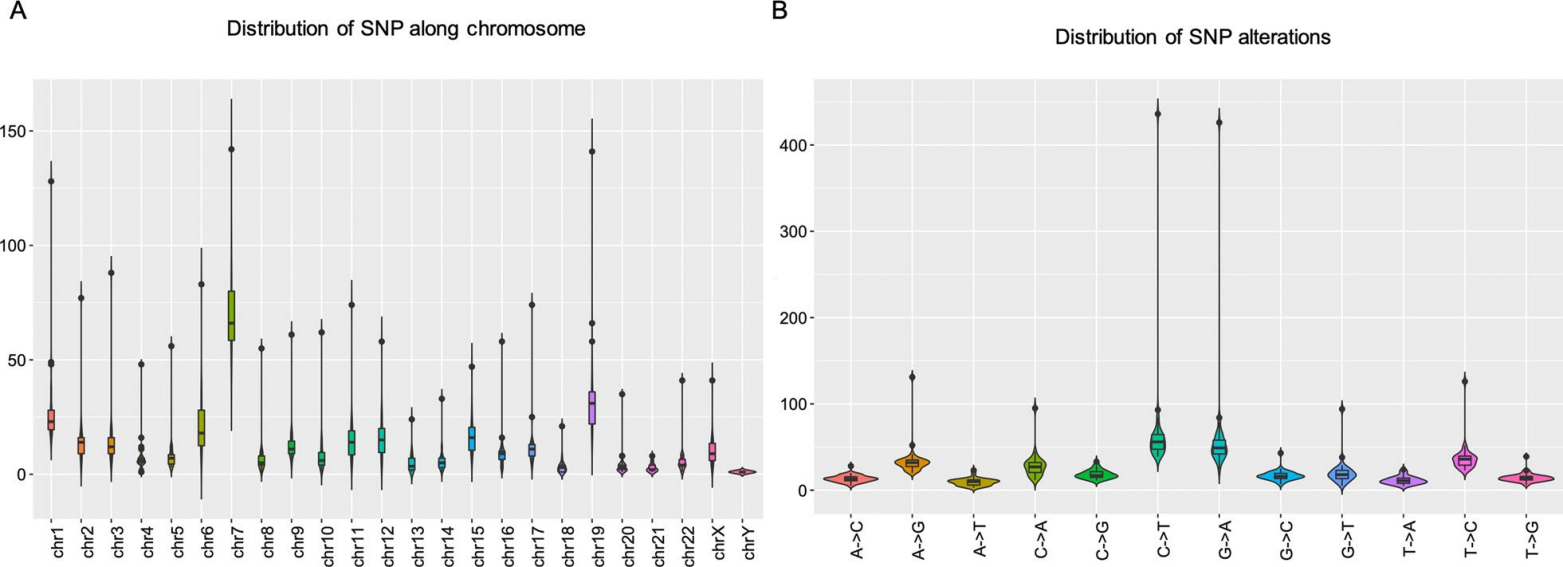
The molecular properties of gastric adenocarcinoma patients collected from Qinghai Provincial People’s Hospital. **A** The distribution of SNP along the chromosome. **B** The distribution of SNP alternation types

As did in our previous work [28], we first checked the sequencing depth at gene level via counting the genome coverage along the gene loci. Most of genes had depth around a hundred no matter in tumor or in normal bio-samples (Additional file 1: Figure S1A, S1C) and genes in tumor bio-samples had lower coverage than in normal bio-samples did (Additional file 1: Figure S1B, S1D). Then we discussed the SNP distribution along the chromosome. The SNP distribution along the chromosome showed that chromosome 7 retained the most of variations (Fig. 2A). According to the database of COSMIC [29], the world's largest and most comprehensive resource for exploring the somatic mutations in human cancer, chromosome 7 included lots of well-known cancer-related genes, including *EGFR, BRAF, CDK6, MET, T1F1*, and so on. The copy number variation in chromosome 7 was also related with cancer [30]. These results together indicated an important region was indicated here. The biomarkers for further diagnosis and treatment could be focus on this important region, that is chromosome 7. The distribution of SNP alteration type showed the C → T and G → A are majority (Fig. 2B). Here, the somatic variation was obtained by using paired normal bio-samples as the reference. Comparing with that obtained by using germline mutation as the reference, the mutation rate became lower (Additional file 1: Figure S1), that might indicate that the paired tumor and normal bio-samples are ideal choice for somatic mutation analysis.

**Fig. 3:**
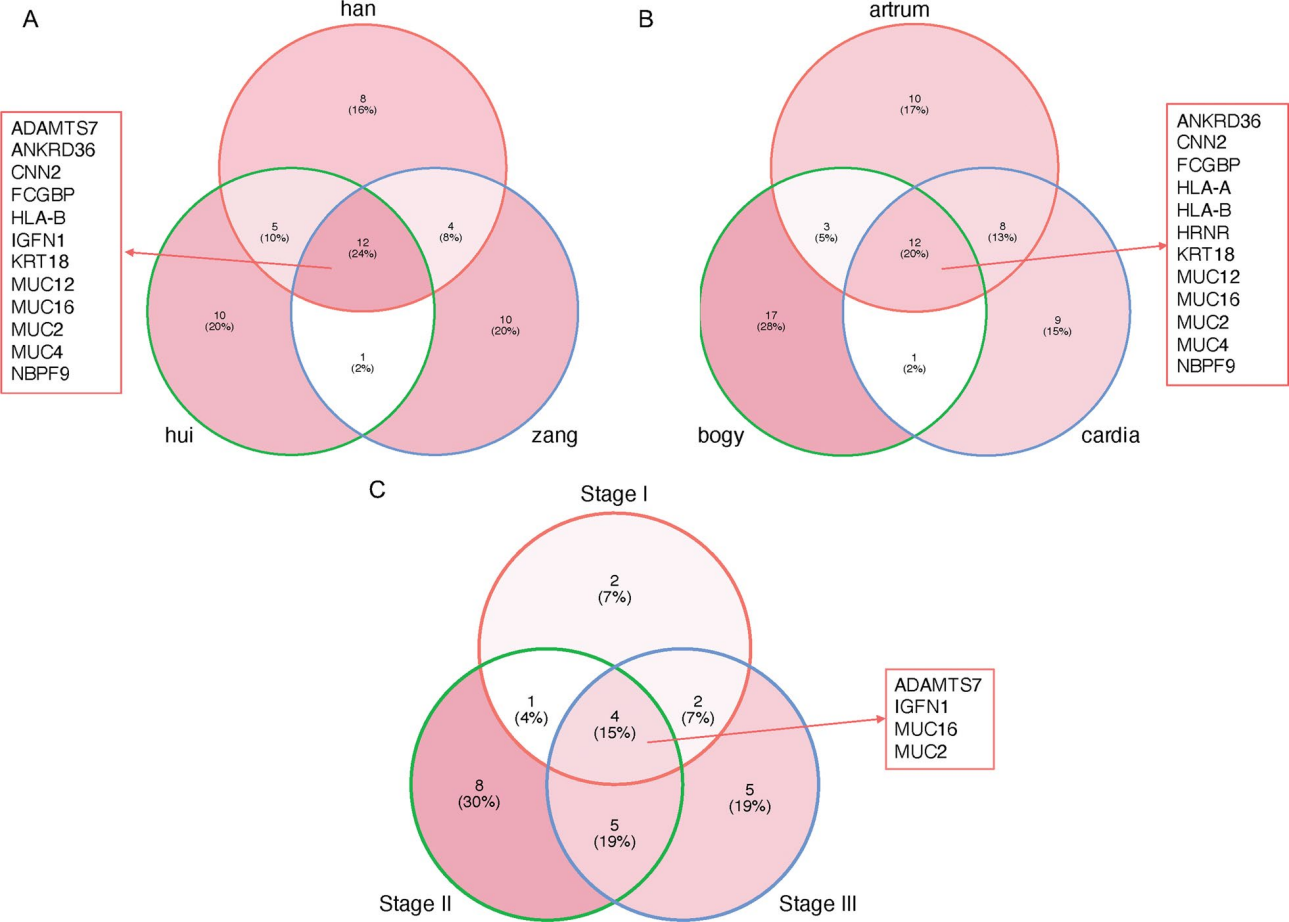
The molecular variance in variance tumor groups. **A** The venn diagram shows the differences in molecular variances between Han Chinese and other minority nationality. **B** The venn diagram shows the differences in molecular variances among various tumor type. **C** The venn diagram shows the differences in molecular variances among various tumor stage

We then linked the molecular variants with clinical features, including patients’ nation, tumor location, and tumor TNM stage. As a result, the venndiagrams showed that various nationality groups, tumor locations, and tumor stages had their unique molecular variants, meanwhile share some common variants. For instance, there were 12 mutant genes share by Han Chinese and other minority nationalities (Fig. 3A), and Han, Hui, and Zang had 8 (*CYP4F2, DSPP, FOXD4, GOLGA6L6, GP6, OR9G1, PABPC3, TBC1D26*), 8 (*ARHGEF26, CNTNAP3B, COL4A2-AS2, ESRRA, LIMS1, OR11H12, PRAMEF22, TPTE, ZNF208, ZNF737*), and 10 (*FAM186A, FOXD4L1, HLA-DPA1, MADCAM1, NBPF11, PCDHA8, POTEG, POTEH, RPGR, ZNF717*) unique mutant genes, respectively; there were 12 mutant genes share by different locations of tumors (Fig. 3B), and antrum, bogy, and cardia had 10 (*AGAP3, ARHGEF26, ARMCX4, CNTNAP3, ESRRA, FLG, GP6, MADCAM1, OTUD7A, PRAMEF22*), 17 (*FBRSL1, FOXD4, FRG2B, GGT2, KCNJ12, MUC17, MUC6, MYO15B, NBPF10, OR2T34, PLIN4, REG3A, RPL21, RPTN, SETD1B, TAS2R20, TPSAB1*), and 9 (*AHNAK2, FMN2, HLA-C, NBPF11, POTEG, RPGR, SLAIN1, VKORC1L1, ZNF208*) unique mutant genes, respectively; there were 4 mutant genes share by different stages of tumors (Fig. 3C), and stage I, stage II, and stage III had 2 (*GOLGA6L6,ZNF717*), 8 (*FAM186A, FMN2, GP6, HLA-C, MAGEC1, PER3, RFPL4A, ZDHHC8*), and 5 (*ANKRD36, HRNR, KRT18, MUC20, MUC6*) unique mutant genes, respectively.

**Fig. 4:**
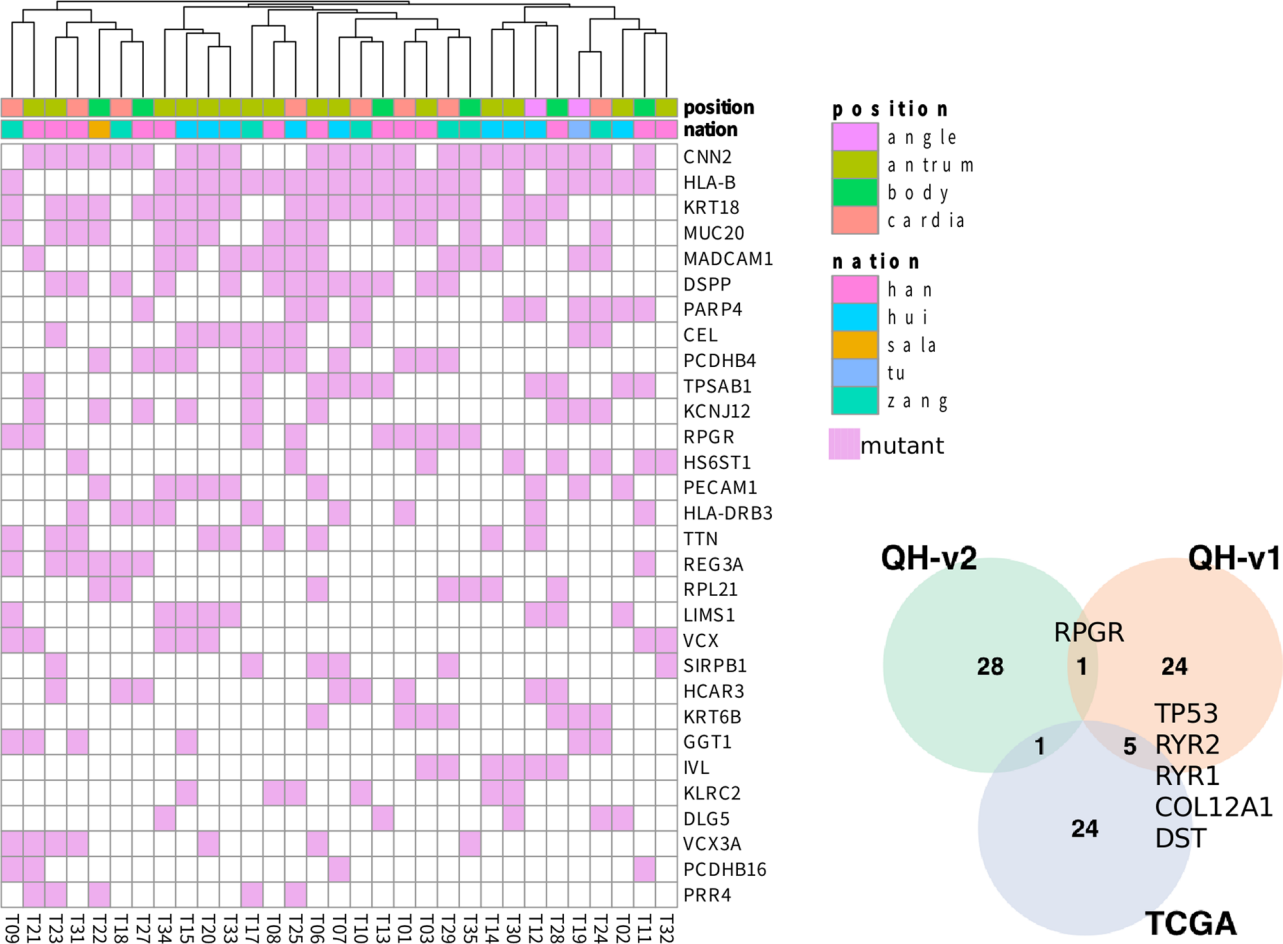
The heatmap shows the cancer driver genes obtained based on MaxMiF in gastric adenocarcinoma patients collected from Qinghai Provincial People’s Hospital. The venn diagram shows the driver genes reported by MaxMiF based on somatic mutations with paired normal variations as reference (QH-v2), germline mutation as reference (QH-v1), and somatic mutations from TCGA patients

To distinguish the potential driver genes from passage genes, MaxMIF was introduced [22]. The first 30 genes with highest MaxMIF scores were reported in Fig. 4. Comparing with using germline mutation as reference (QH-v1), only *RPGR* was shared, meaning the reference played very important role in determining the somatic mutation (Fig. 4). Also, only five genes, *TP53, RYR2,RYR1, COL12A1, DST*, were also presented in western patients (Fig. 4). Furthermore, from Fig. 4, we can see that, the mutation profiles for these driver genes were not shown the significant differences between Han Chinese and other minor ethnic groups. The follow-up gene fusion analysis showed in-frame gene fusion of the number 2 exon of *KRTAP10-7* and number 1 exon of *KRTAP10-6* (chr21:46020997 → chr21: 46011685), and number 28 exon of *IPO4* and number 21 exon of *DNHD1* (chr14:24650800 → chr11: 6567900), which were also found by using germline mutation to determine the somatic mutation. Besides that, it also identified some unique fusion genes, including the in-frame gene fusion of the number 1 exon of *HOXD11* and number 11 exon of *AGAP3* (chr2:176972342 → chr7: 150783926), and in-frame gene fusion of the number 8 exon of *HLA-A* and number 7 exon of *HLA-J* (chr6:29913277 → chr6: 29977361).

In sum, the unique clinical and molecular characteristics for plateau patients were detected, meaning that there should have some treatment strategies that are specifically designed for them.

### Prediction of effective clinical anti-cancer drug for gastric patients living in QTB

**Fig. 5:**
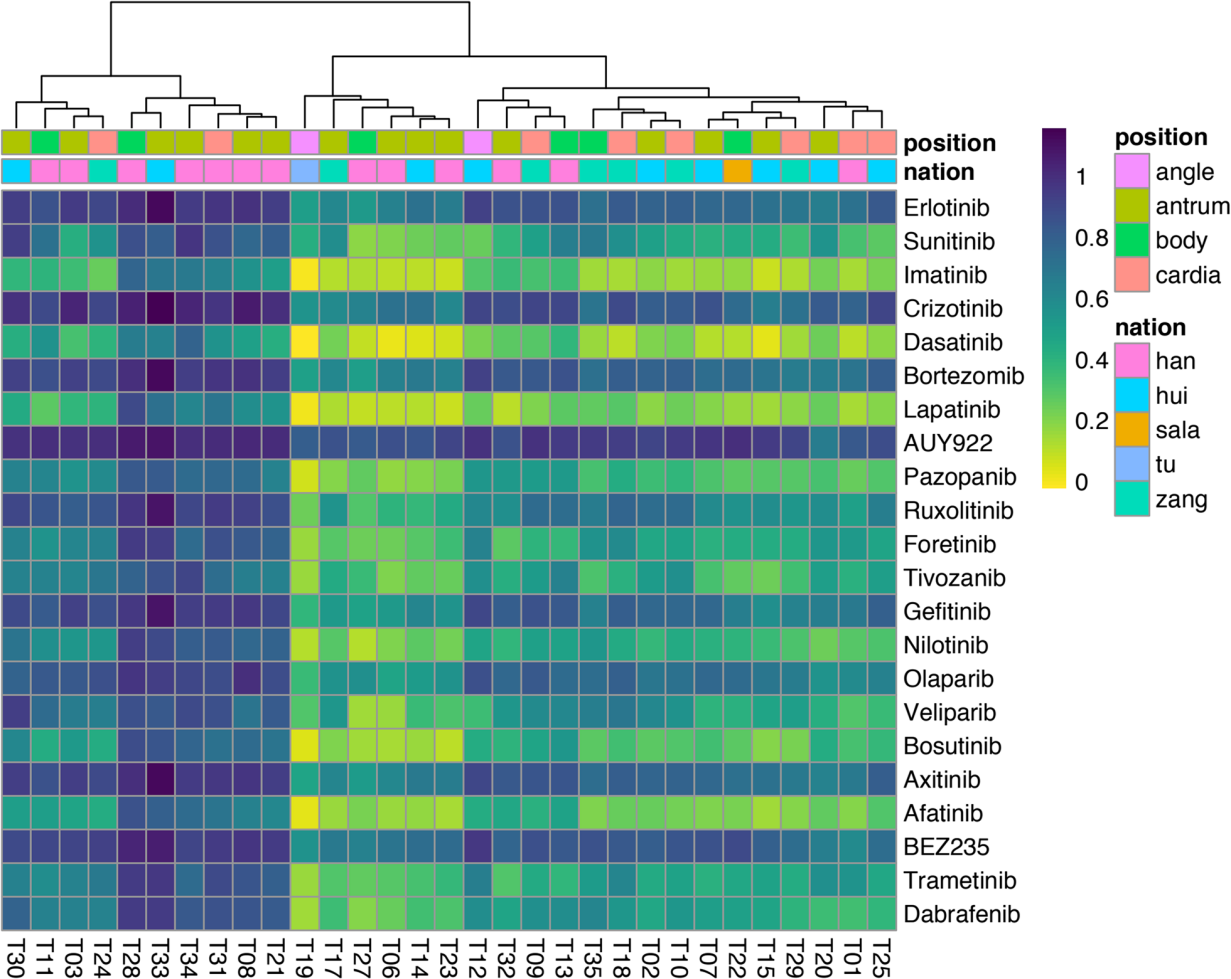
The predicted effective clinical anti-cancer drug for gastric adenocarcinoma patients collected from Qinghai Provincial People’s Hospital. The ethnic group and the tumor location are shown with different colors

Thus, to detect the effective clinical anti-cancer drugs for these patients living in QTB, we trained our previously adapted kernel-based learning model [19] on pharmacogenomics generated from Genomics of Drug Sensitivity in Cancer [31], to predict the effective clinical drugs for these patients. The predicted results showed that there were no significant differences between Han Chinese and minor ethnic groups in terms of effective clinical drugs (Fig. 5), which may be due to the fact that Han Chinese and minor ethnic groups of patients did not have significant differences in molecular characteristics. All 31 patients had responsed well to six drugs, including Erlotinib (EGFR inhibitor), Crizotinib (Met inhibitor), Bortezomib (Proteasome inhibitor), AUY922 (Hsp90 inhibitor), Axitinib (VEGFR inhibitor), and BEZ235 (PI3K inhibitor). There were few reports of Crizotinib in gastric cancer patients with c-MET amplification [32]. Therefore, it might be a good option for that patients living in QTP.

## Discussion

Qinghai province is in the northeast of QTP with average 4000-m height above sea level. Han Chinese and lots of minor ethnic groups (such as hui, zang (Tibetan people), zhi, sala, and so on) lived here. In addition, people living here always have alcohol and cigarette issue. The gastric cancer, which are related with dietary and lifestyles, is the most common cancer types in Qinnghai province [28]. Here, to better understand the molecular mechanism of gastric cancer patients living in QTP, and predict the most effective clinical drugs for these patients, we collected the paired tumor and normal bio-samples from gastric patients at Qinghai Provincial People’s Hospital, and discussed the clinical and molecular characteristics for those patients. As a result, we found some unique characteristics for our plateau patients, including has lower mutation rate, and unique gene fusions. The drug response prediction mode based on pharmacogenomics from GDSC suggests the effective targeted therapies, which specifically designed for these plateau patients. The prediction results could be evaluated by following up tracking reports.

There are around 5,000,000 people living in Qinghai province, and based on the recent statistic reports, only around 1,000,000 people live in Xining. The sample collection and followed up tracing are quite challenge here. It took us two years to collect these 30 patients. In future, we will collect much more patients with followed up treatment reports to further show the unique characteristics of plateau patients and discuss their special treatment strategy.

## Conclusion

Here, to better understand the molecular mechanism of gastric cancer patients living in QTP, and predict the most effective clinical drugs for these patients, the WES was performed on tumour and paired normal bio-samples from 31 primary gastric cancer patients at Qinghai Provincial People’s Hospital. Several unique molecular characteristics for those gastric cancer patients were found, including having more SNPs located in chromosome 7 with C → T and G → A as the most common alteration types, barely sharing the cancer driver genes with western patients, and having no significant differences in various Chinese nation, et al.

## Supplementary Information

**Additional file 1.** The supplementary figures and tables.

## Data Availability

The datasets used and/or analysed during the current study available at https://www.jianguoyun.com/p/DeRPAeYQj7eWChi22qUE.
