# Supplementary material for: The molecular characteristics of gastric cancer patients living in Qinghai-Tibetan Plateau
Source: BMC Gastroenterol. 2022 May 14;22:244. doi: 10.1186/s12876-022-02324-8 (PMC9107197; doi:10.1186/s12876-022-02324-8)

Supporting information

**The molecular characteristics of gastric cancer patients living in Qinghai-Tibetan Plateau**

LingYuan^1,2^, Shilong Chen^3,4^, Yongcui Wang^3,4*^, Yingcai Ma^2*^

^1^ [Department](javascript:;) [of](javascript:;) [Medicine](javascript:;), Soochow University, Suzhou, Jiangsu, 2115006, China

^2^ Department of Gastroenterology, Qinghai Provincial People’s Hospital, Xining, Qinghai, 810007, China

^3^ Institute of Sanjiangyuan National Park, Chinese Academy of Sciences, Xining, 810008, China

^4^ Qinghai Provincial Key Laboratory of Crop Molecular Breeding, Northwest Institute of Plateau Biology, Chinese Academy of Sciences, Xining, 810008, China

* To whom correspondence should be addressed.

Email: mayingcai0271@sina.com; ycwang@nwipb.cas.cn

^#^ These authors contributed equally to this work as first authors.

Legend

Table S1.

The details of fusion genes obtained by FusionMap.

**Figure S1.** The distribution of genome coverage at gene level in gastric adenocarcinoma patients collected from Qinghai Provincial People’s Hospital. A, B, the distribution of genome coverage at gene level in tumor bio-samples, C,D, the distribution of genome coverage at gene level in normal bio-samples.

Figure S2

The comparison of mutation rates in terms of somatic variation obtained via different references.

Table S1

| **No. of patients with fusion gene reports** | **Fusion gene (exon number)** | **position** | **FrameShiftClass** |
| --- | --- | --- | --- |
| 9 | IPO4 (28)->DNHD1 (21) | chr14:24650800🡪chr11: 6567900 | InFrame |
| 6 | HOXD11 (1)🡪AGAP3 (11) | chr2:176972342🡪chr7: 150783926 | InFrame |
| 5 | HLA-A (8)🡪HLA-J (7) | chr6:29913277🡪chr6: 29977361 | InFrame |
| 2 | KRTAP10-7 (2)->KRTAP10-6 (1) | chr21:46020997🡪chr21: 46011685 | InFrame |

Figure S1.

Figure S2


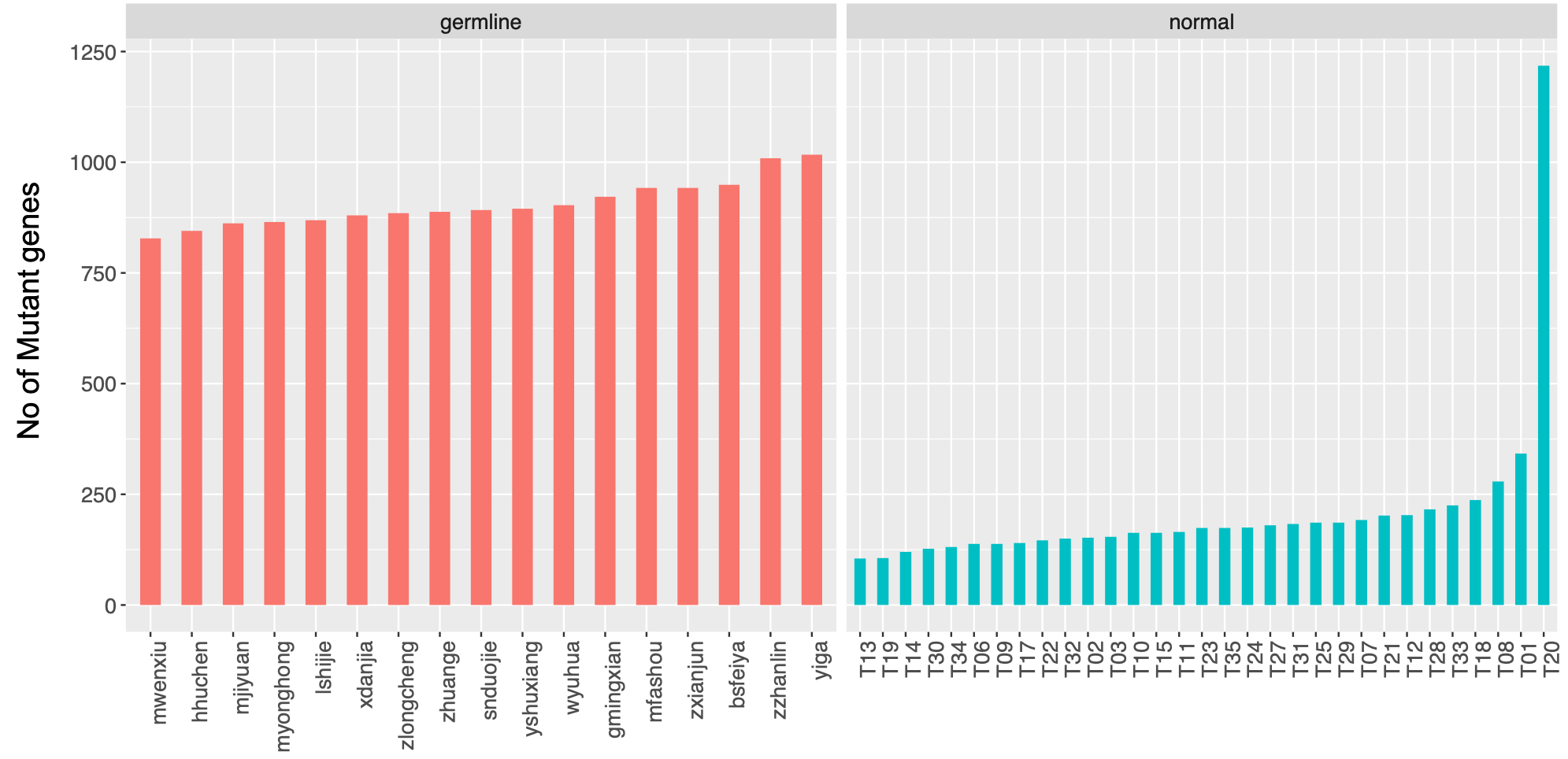

Supplement: Supplementary file 1 — Additional file 1. The supplementary figures and tables. [file 12876_2022_2324_MOESM1_ESM.docx]
